# Effect of Subsoiling in Fallow Period on Soil Water Storage and Grain Protein Accumulation of Dryland Wheat and Its Regulatory Effect by Nitrogen Application

**DOI:** 10.1371/journal.pone.0075191

**Published:** 2013-10-02

**Authors:** Min Sun, ZhiQiang Gao, WeiFeng Zhao, LianFeng Deng, Yan Deng, HongMei Zhao, AiXia Ren, Gang Li, ZhenPing Yang

**Affiliations:** College of Crop Science, Shanxi Agricultural University, Taigu, Shanxi Province, China; Nanjing Agricultural University, China

## Abstract

To provide a new way to increase water storage and retention of dryland wheat, a field study was conducted at Wenxi experimental site of Shanxi Agricultural University. The effect of subsoiling in fallow period on soil water storage, accumulation of proline, and formation of grain protein after anthesis were determined. Our results showed that subsoiling in fallow period could increase water storage in the 0–300 cm soil at pre-sowing stage and at anthesis stage with low or medium N application, especially for the 60–160 cm soil. However, the proline content, glutamine synthetase (GS) activity, glutamate dehydrogenase (GDH) activity in flag leaves and grains were all decreased by subsoiling in fallow period. In addition, the content of albumin, gliadin, and total protein in grains were also decreased while globulin content, Glu/Gli, protein yield, and glutelin content were increased. With N application increasing, water storage of soil layers from 20 to 200 cm was decreased at anthesis stage. High N application resulted in the increment of proline content and GS activity in grains. Besides, correlation analysis showed that soil storage in 40–160 cm soil was negatively correlated with proline content in grains; proline content in grains was positively correlated with GS and GDH activity in flag leaves. Contents of albumin, globulin and total protein in grains were positively correlated with proline content in grains and GDH activity in flag leaves. In conclusion, subsoiling in fallow period, together with N application at 150 kg·hm^−2^, was beneficial to increase the protein yield and Glu/Gli in grains which improve the quality of wheat.

## Introduction

Natural precipitation is the only water resource of dryland wheat. Rainfalls always concentrate from July to September at region producing dryland wheat in loess plateau, which is called fallow period. During the fallow period, soil water is replenished and restored. It is the water stored in this period that determines the soil moisture before dryland wheat is sown which has a great influence on the production of dryland wheat [1]–[4]. In these days, though great progress has been made in the technology of water storage and retention in dryland cultivation [5]–[8], water storage and retention in fallow period had been ignored. Therefore, finding an effective way to raise the moisture content in fallow period soil is significant to the dryland wheat production.

Many researches showed protein content in wheat grains would increase when the water was deficient in soil [9]–[13]. For example, Sun et al. (2010) showed that cultivation in dry land could increase the content of albumin, gliadin, glutetin, total protein and Glu/Gli in grains while decrease the content of glublin [11]. Zhao et al. (2007) found that drought in milking stage increased the content of protein and Glu/Gli in grains [9]. Fan et al. (2004) also reported drought in milking stage could increase Glu/Gli [12]. In addition, Fu et al. (2008) suggested drought in milking stage combined with nitrogen application could increase protein and nitrogen content in wheat grains [10]. Content and component of grain protein, especially for the Glu/Gli, not only influence the nutrition quality of wheat, but also influence the processing quality of wheat [13].

Up to date, problems in production of wheat in dry land were enumerated as follows: 1) Low water-using efficiency caused by poor soil fertility and low ability of soil water storage and retention; 2) Late straw rotting fails to increase the organic matter of soil, and thus, affect the quality of sowing; 3) Although precipitation in fallow period is considerably abundant, a great evaporation loss of surface water fails to store enough water for spring because of the high temperature in summer; 4) Wheat is often sown under enough moisture or rainfall in dry land, so the sowing time is inconsistent. Many researches showed drought can improve the quality of wheat to a certain degree [14]–[16], [10], [9], [17]. Thus, the wheat quality should be taken into account as the level of water storage raised in the fallow period.

The objective of the present paper was to provide a new way to increase the water storage and retention for dryland wheat. This was realized by subsoiling after wheat harvest and conducting rotary tillage in the late August. The proline content and physiological mechanism about formation of grain protein in dryland wheat were examined. To have a comprehensive understanding for the issue of dryland wheat quality, the effects of fertilizer application were also studied.

## Materials and Methods

The field experiment was conducted at Wenxi experimental site (summer fellow) of Shanxi Agricultural University from 2009 to 2011. The soil fertility was measured on 1^st^ July, 2010. Content of organic matter was measured as 8.65 g/kg using potassium dichromate volumetric method. Total nitrogen was measured as 0.74 g/kg using Kjeldahl determination, alkali-hydrolyzable nitrogen was measured as 32.93 mg/kg using alkali-hydrolyzable proliferation method, and rapidly available phosphorus was measured as 20.08 mg/kg using sodium bicarbonate method.

The experimental site is very arid and 60%–70% of the annual precipitation is concentrated in summer and early-fall (July, August, and September). Precipitation conditions at the experimental site in 2005–2011 were shown in Table 1. These data were provided by Agricultural Bureau of Wenxi County. Annual precipitation in 2010–2011 was a little higher than conventional years and the precipitation in fallow period was higher than early growth stage. The precipitation in fallow period averagely accounted for 58% of annual precipitation in 2005–2011 and 75% in 2010–2011 (Table 1).

**Table 1:** Precipitation at the experimental site in Wenxi (mm).

			Pre-wintering	Elongation		
Year	Fallow period	Sowing-Pre-wintering	–	–	Anthesis-Mature	Total
			Elongation	Anthesis		
2005–2011 (average)	291	63	41	34	76	505
2010–2011	402	27	19	22	64	534

Fallow period (The first ten days of Jul. to last ten days of Oct.);

Sowing-Pre-wintering (The first ten days of Oct. to last ten days of Nov.);

Pre-wintering- Elongation (The last ten days of Nov. to first ten days of Apr.);

Elongation -Anthesis (The first ten days of Apr. to first ten days of May);

Anthesis-Mature (The first ten days of May to middle ten days of Jun).

### Design of Field Experiment

The experiment was laid out as split-split plot arrangement and three replications. Two levels (subsoil (SS); and no-tillage (CK)) were allocated to the main plots. Sub plots were three levels of N fertilizer application (75 kg·hm^−2^ (LN), 150 kg·hm^−2^ (MN) and 225 kg·hm^−2^ (HN)). For subsoil plot, the preceding wheat residues were shredded on the fifth day after harvest (i.e. July 1^st^, 2010), followed by tillage that consisted of disking 30–40 cm deep before planting wheat. Then soil moisture was retained by rotary tillage on 20^th^ August, 2010.

Dryland wheat cultivar Yunhan 20410, which provided by agricultural bureau of wenxi county, was planted on 29^th^ September, 2010 with a row spacing of 20 cm and a seeding rate of 225×10^4^/hm^2^. The area of experimental site was 300 m^2^ (50 m in length and 6 m in width). Phosphate and potash fertilizer had been applied to the field with P_2_O_5_ and K_2_O at a concentration of 150 kg·hm^−2^.

### Sampling and Measurement Methods

#### Measurement of soil water storage

Soil water storage was measured by drying method. Soil samples in the 0–300 cm depth (20 cm per soil layer) were obtained at pre-sowing stage, pre-wintering stage, seedling establishment stage, elongation stage, booting stage, anthesis stage, and maturation stage using a soil auger. The calculation formula of soil water storage is as follows:

Soil water storage (mm) = [(mass of damp soil − mass of dried soil)/mass of dried soil×100]×soil thickness (mm)×volume weight per layer.

#### Measurement of proline and activity of nitrogen metabolism enzyme in flag leaves and grains

Wheatears with consistent growth and blooming on the same day were tagged at anthesis stage and fifteen ears were sampled every five days after anthesis. Flag leaves and grains were kept at −40°C after being quickly frozen by liquid nitrogen for measuring the activity of enzyme. Another five fresh samples, i.e. flag leaves and grains, were taken to measure proline content. The measurement of proline content was referred to [18] and activity of glutamine synthetase (GS) and glutamate dehydrogenase (GDH) were measured according to the method of [19].

#### Measurement of grain protein and its components

Wheatears with consistent growth and blooming on the same day were tagged at anethsis stage and fifteen ears were sampled every five days after anethsis. Grains were separated in the oven where moisture was removed by heating at 105,°C for 30 min and then weighted after drying at 80°C. Grains were used to measure the content of protein and its component after being crushed by micro high-speed universal grinder. Nitrogen content was measured using the method of semi micro Kjeldahl determination and wheat protein content was obtained by multiplying N content (%) by 5.7 [20]. Content of albumin, globulin, gliadin, and glutelin in grains were measured using the method of continuous extraction.

#### Measurement of grain yield

Number of grains ears per unit area, average number of grains per wheat ear, and 1000-grain weight were investigated at maturation stage. Fifty plants of wheat were taken from each plot to measure biological yield, and twenty square meters of wheat were rope to measure economical yield.

### Statistical Analysis

Statistical analysis was conducted using SAS version 8.0 (1999, SAS Institute, Inc., Cary, NC, USA). Analysis of variance (ANOVA) using the General Linear Model procedure and the difference between means using the Duncan test were determined at the α level of 0.05.

## Results

### Effect of Subsoiling in Fallow Period on Soil Water Storage and its Regulatory Effect by Nitrogen Application

#### Effect on soil water storage before sowing

**Figure 1:**
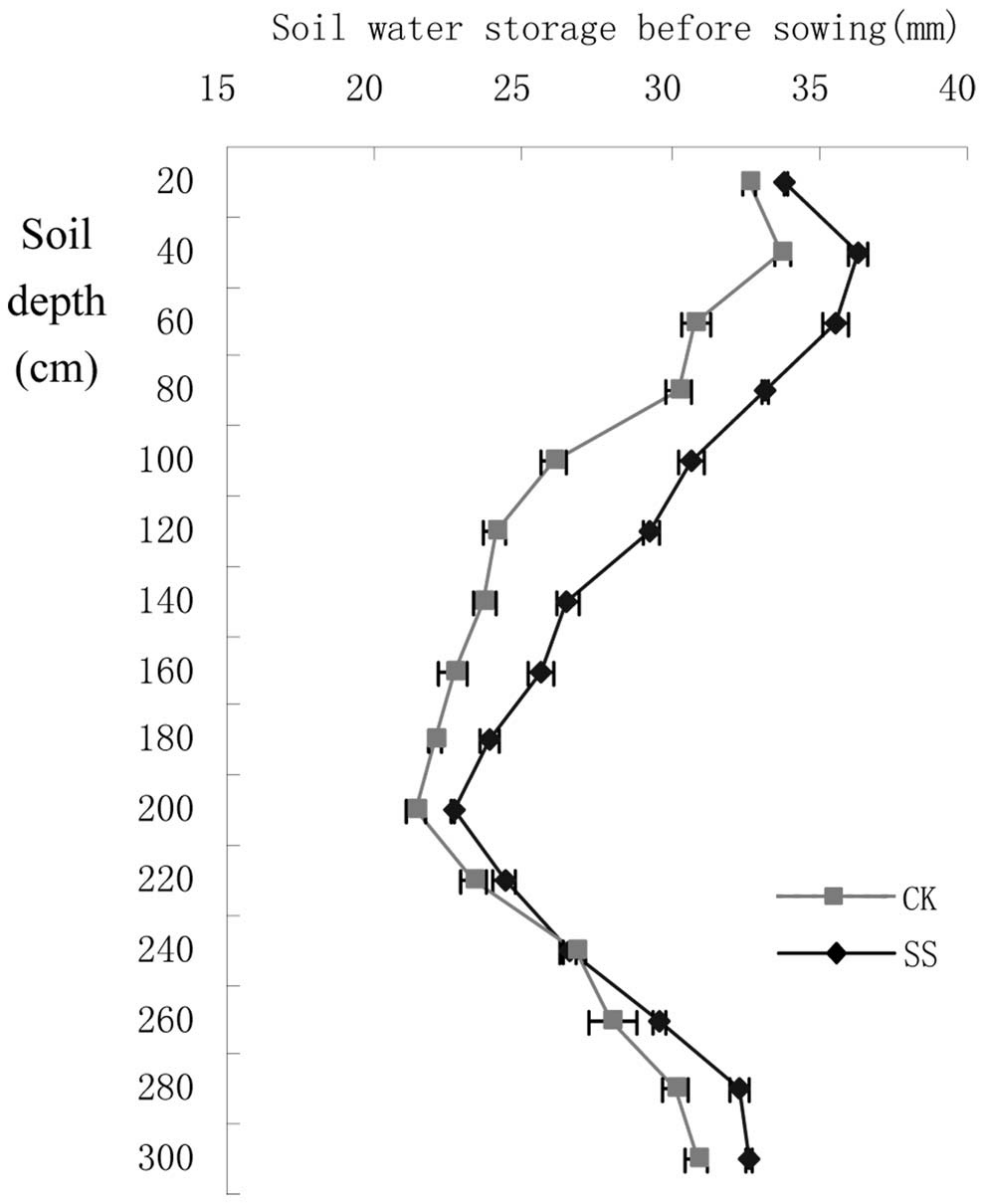
Soil water storage in SS were increased in the depth of 0–300 cm compared with CK. SS, Subsoiling; CK, no-tillage.

Generally, as the depth of soil increasing, water storage in the 0–300 cm depth soil before sowing increased at first but then decreased to the minimum at 200 cm and finally tended to increase again (Fig. 1). Compared with CK, soil water storage in SS were increased by 16.08 mm, 14.21 mm, and 6.23 mm in the depth of 0–100 cm, 100–200 cm, and 200–300 cm, respectively. The increment in the 0–300 cm depth reached to 36.25 mm in total and the increment of the 60–160 cm depth soil account for more than 10% of the whole storage. Accordingly, subsoiling in fallow period could significantly increase water storage and retention which provided favorable conditions for proper sowing of dryland wheat.

#### Effect on soil water storage in anthesis

Generally, as the depth of soil increasing, water storage of soil in anthesis in the 0–300 cm depth increased to maximum at 80 cm, and then decreased between 80 cm to 120 cm, and finally tended to increase slowly again (Table 2). Table 2 showed that, under low and medium N application, subsoiling in fallow period could increase water storage at each layer of soil with the depth of 0–200 cm (20 cm per layer). The difference of water storage between SS and CK was statistically significant in the depth of 80, 200, 260, and 300 cm under low N application and in the depth of 20–40 cm and 80–180 cm under medium N application. Compared with CK, subsoiling in fallow period could also increase water storage of soil layers in the 240–300 cm depth (20 cm per layer) under low N application.

**Table 2:** Effect of subsoiling in fallow period on soil water storage in anthesis and its regulation by nitrogen application(mm).

Tillage	N applicationamount	soil layer(cm)						
		0–100					0–100	
		20	40	60	80	100		
SS	LN	14.12a	18.14a	20.25a	20.54a	19.45a	92.50a	
	MN	12.58b	17.01b	19.19b	20.31a	18.77a	87.85ab	
	HN	12.18b	15.41c	17.54c	18.24b	15.65b	79.02b	
CK	LN	14.01a	17.68a	19.84a	19.64a	19.02a	90.19a	
	MN	11.08c	15.92b	19.06ab	19.62a	17.63b	83.31b	
	HN	12.21b	15.52b	18.41b	18.98a	16.85c	81.97b	
		**100–200**					**100–200**	**0–200**
		**120**	**140**	**160**	**180**	**200**		
SS	LN	19.50a	20.12a	21.05a	21.45a	22.15a	104.27a	196.77a
	MN	18.40b	19.24b	19.98b	20.02b	20.34b	97.97ab	185.83b
	HN	16.52c	17.98c	18.02c	18.95c	18.24c	89.71b	168.73c
CK	LN	19.02a	19.87a	20.85a	21.12a	21.25a	102.11a	192.30a
	MN	17.16b	18.19b	19.24b	19.60b	20.03b	94.21b	177.53b
	HN	17.02b	17.92b	18.96b	19.35b	19.58b	92.83b	174.80b
		**200–300**					**200–300**	**0–300**
		**220**	**240**	**260**	**280**	**300**		
SS	LN	21.25a	22.02a	23.25a	24.12a	25.68a	116.32a	313.09a
	MN	20.62a	21.06b	21.20c	22.57b	22.83c	108.28a	294.11b
	HN	20.98a	21.54ab	22.24b	23.75a	23.98b	112.49a	281.22c
CK	LN	21.02a	21.98a	22.57a	23.65a	24.52a	113.74a	306.04a
	MN	20.66a	20.91b	21.13b	22.08b	22.69b	107.46ab	284.99b
	HN	19.85b	20.25b	20.58b	21.68b	22.08b	104.44b	279.24b

The difference between data with different letters from a to c are statistically significant (P<0.05).

SS: subsoiling; CK: no tillage; LN: low nitrogen application; MN: medium nitrogen application; HN: high nitrogen application.

Table 2 also indicated that the water storage in the 0–200 cm depth soil under the condition of subsoiling tended to decrease as the nitrogen application increasing and so did the water storage in the depth of 20–300 cm soil in the control. Moreover, soil water storage in the depth of 40 cm, 60 cm and 120–200 cm varied significantly among different treatments (three levels of N application) under the condition of subsoiling. Also, the maxium soil water storage in the 200–300 cm depth was obtained with low nitrogen application while the minimum was obtained with medium nitrogen application under the same condition. And soil water storage in the depth of 40 cm, 100–200 cm and 240–300 cm with low nitrogen application were significantly higher than that with medium and high nitrogen application under control conditions.(Table 2).

### Effect of Subsoiling in Fallow Period on Proline Content after Anthesis and its Regulatory Effect by Nitrogen Application

**Figure 2:**
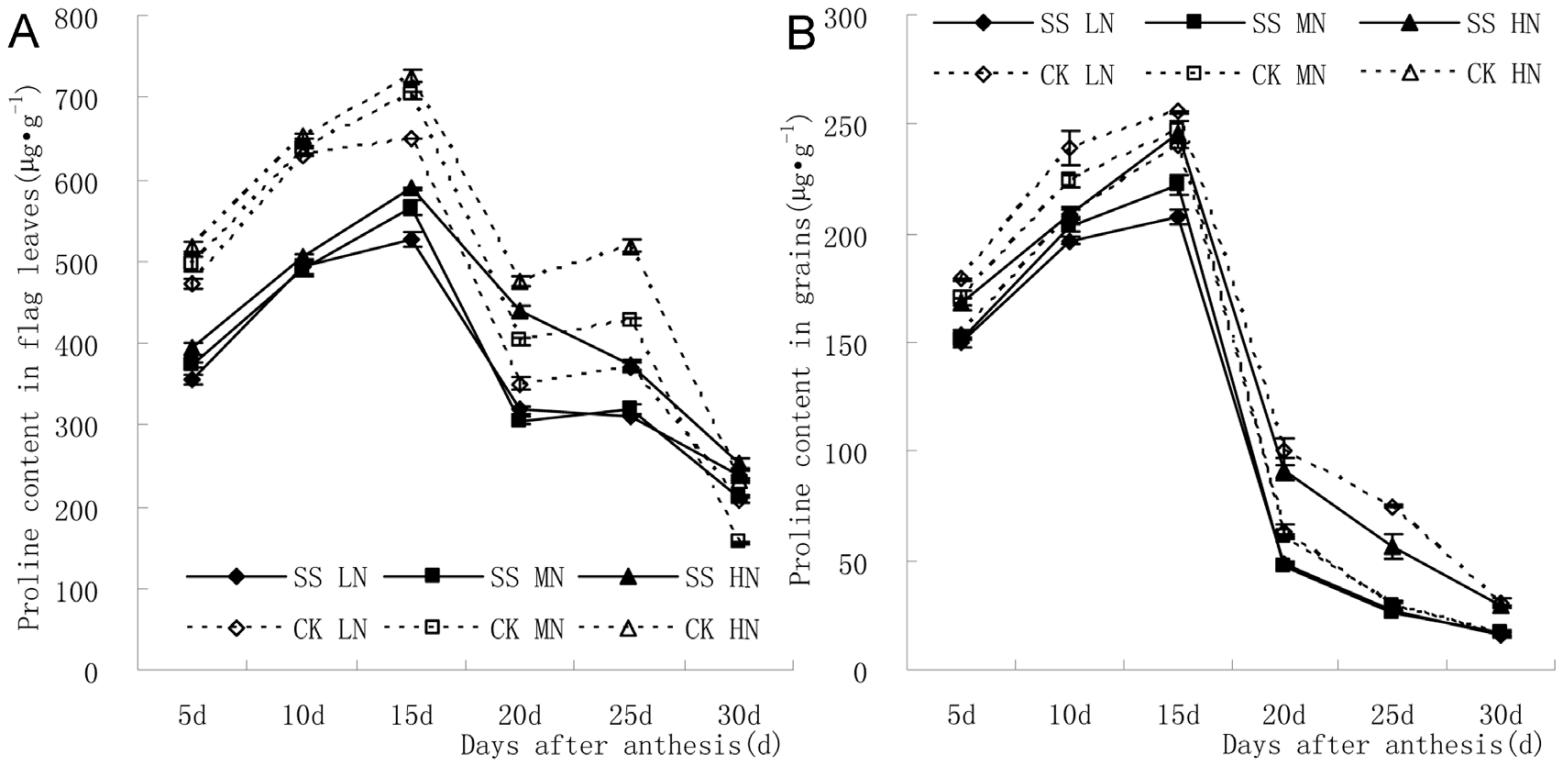
Proline content in flag leaves presented a “M”-type change tendency (A). and proline content in grains displayed a unimodal curve and the only peak appeared at 15d after anthesis (B).

Fig. 2 displays the changes of proline content in flag leaves and grains after anthesis. In all treatments, proline content in flag leaves presented a “M”-type change tendency. Two peaks respectively appeared at 15 d and 20 d after anthesis and the former one was higher. On the other side, proline content in grains displayed a unimodal curve and the only peak appeared at 15d after anthesis.

Fig. 2A indicated that subsoiling in fallow period significantly decreased proline content in flag leaves after anthesis. Under the condition of subsoiling, the increase of nitrogen application led to increase of proline content in flag leaves at 5–15 d and 25 d after anthesis. The proline content was significantly higher at 5 d and 15–25 d after anthesis with high N application. Proline content in flag leaves at 5–25 d in the control was also increased. Proline content in flag leaves varied significantly among different N application treatments at 10–25 d after anthesis. Moreover, the regulatory effect of N application on proline content in flag leaves was more significant in subsoiling plot than that in no-tillage plot at the medium and late milking stage.

Fig. 2B showed that proline content in grains was decreased by subsoiling in fallow period and the difference at 10–20 d after anthesis with low N application, 5–20 d with medium N application, and 5–15 d, 25 d with high N nitrogen application were all statistically significant. In subsoiling treatment, the increase of nitrogen application increased proline content in grains at 5–15 d and 30 d after anthesis. Proline contents in grains at 20 d and 25 d after anthesis were the highest in high N application treatment and the lowest in medium N application treatment, respectively. However, the difference between medium and low N application treatments were not significant. Additionally, the regulatory effect of N application on proline content in grains was more significant in subsoiling plot than that in no-tillage plot at the early and late milking stage.

### Effects of Subsoiling in Fallow Period on Activity of Nitrogen Metabolism Enzyme and its Regulatory Effect by Nitrogen Application after Anthesis

**Figure 3:**
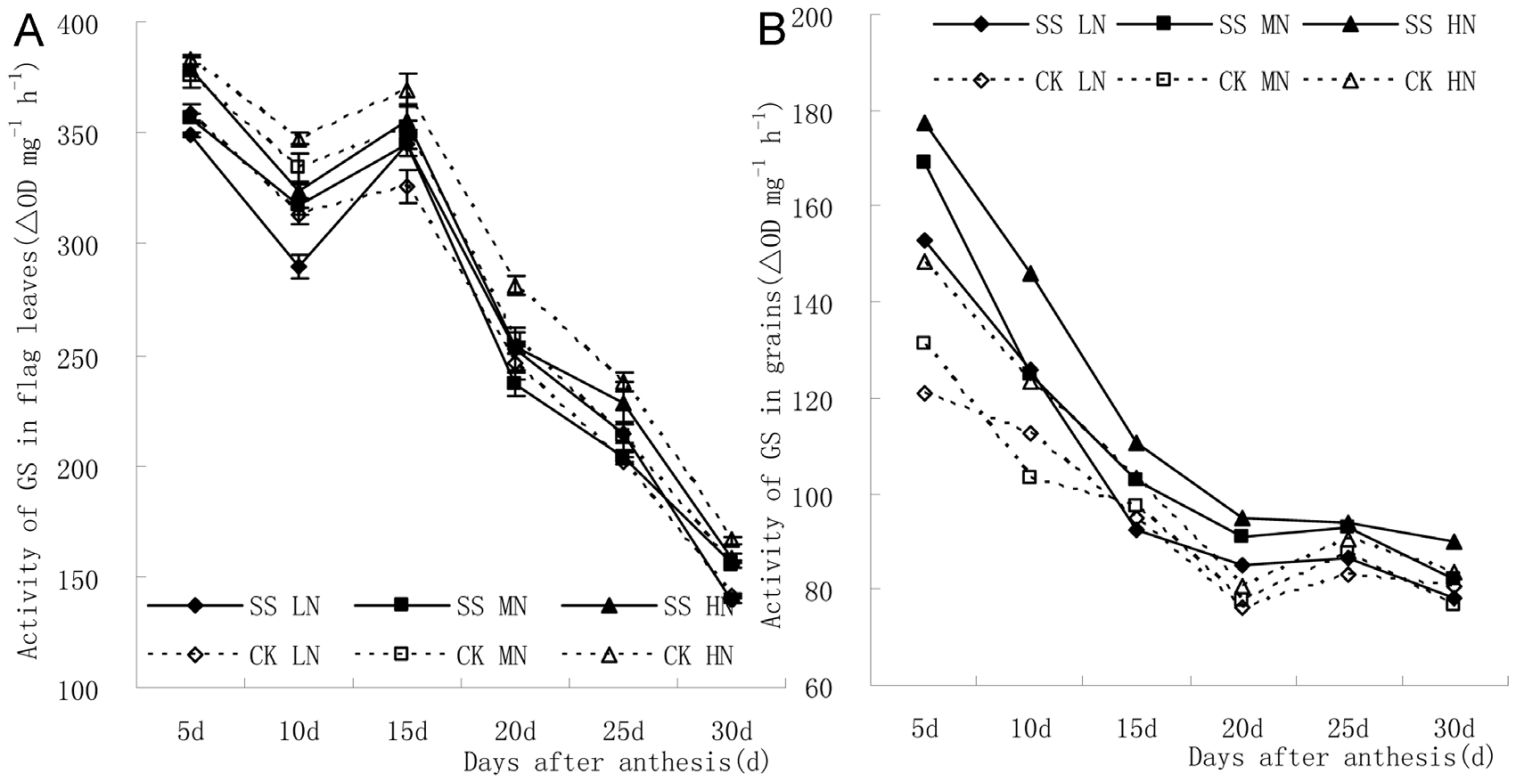
The GS activity in flag leaves decreased at first and then increased until 15(A). The GS activity in grains decreased until 20(B).

Fig. 3 displays the change tendency of GS activity in flag leaves and grains after anthesis. Generally, the GS activity in flag leaves decreased at first and then increased until 15 d after anthesis, and it rapidly decreased again. The GS activity in grains decreased until 20 d after anthesis, and then it tended to level off.

Fig. 3A indicated that GS activity in flag leaves was decreased by subsoiling in fallow period with medium and high N application. The GS activity in subsoiling plot at 5 d, 10 d, and 20 d after anthesis with medium N application, and at 10–20 d and 30 d with high N application were significantly decreased compared with that in no-tillage plot. The GS activity in subsoiling plot was decreased at 15–25 d with low N application, but only that at 15 d after anthesis was significantly different. Under subsoiling condition, the increase of N application increased GS activity in flag leaves at 5 d, 10 d and 30 d after anthesis, and the difference between high N application and low N application was significant. In addition, GS activity in flag leaves in no-tillage plot was also increased along with the increase of N application, and the difference between low N application and other treatments at 5–15 d after anthesis were significant. So was the difference between high N application and other treatments at 20–30 d after anthesis. Under the condition of subsoiling, the maxium GS activity in flag leaves at 15–25 d was observed in high N application treatment while the minimum was observed in medium N application treatment. However, the difference among the three N application treatments were not statistical significant. In addition, the regulatory effect of N application on GS activity in flag leaves was more significant in subsoiling plot than that in no-tillage plot at the medium and late milking stage.

Fig. 3B showed that subsoiling in fallow period could increase GS activity in grains in almost all three N application treatments (except at 15 d after anthesis with low N application). And the difference of GS activity between subsoiling plot and no-tillage plot were all statistically significant except for that at 15 d with medium N application and at 25 d with high N application. Increment of N application increased GS activity in grains except for the 15 d sample after anthesis. Besides, the GS activity in low N application treatment was significantly different from other treatments at 15–20 d after anthesis, while the difference between medium and high N treatments were not significant. In the no-tillage plot, the GS activity between high N application and other treatments were significantly different (Fig. 3B).

**Figure 4:**
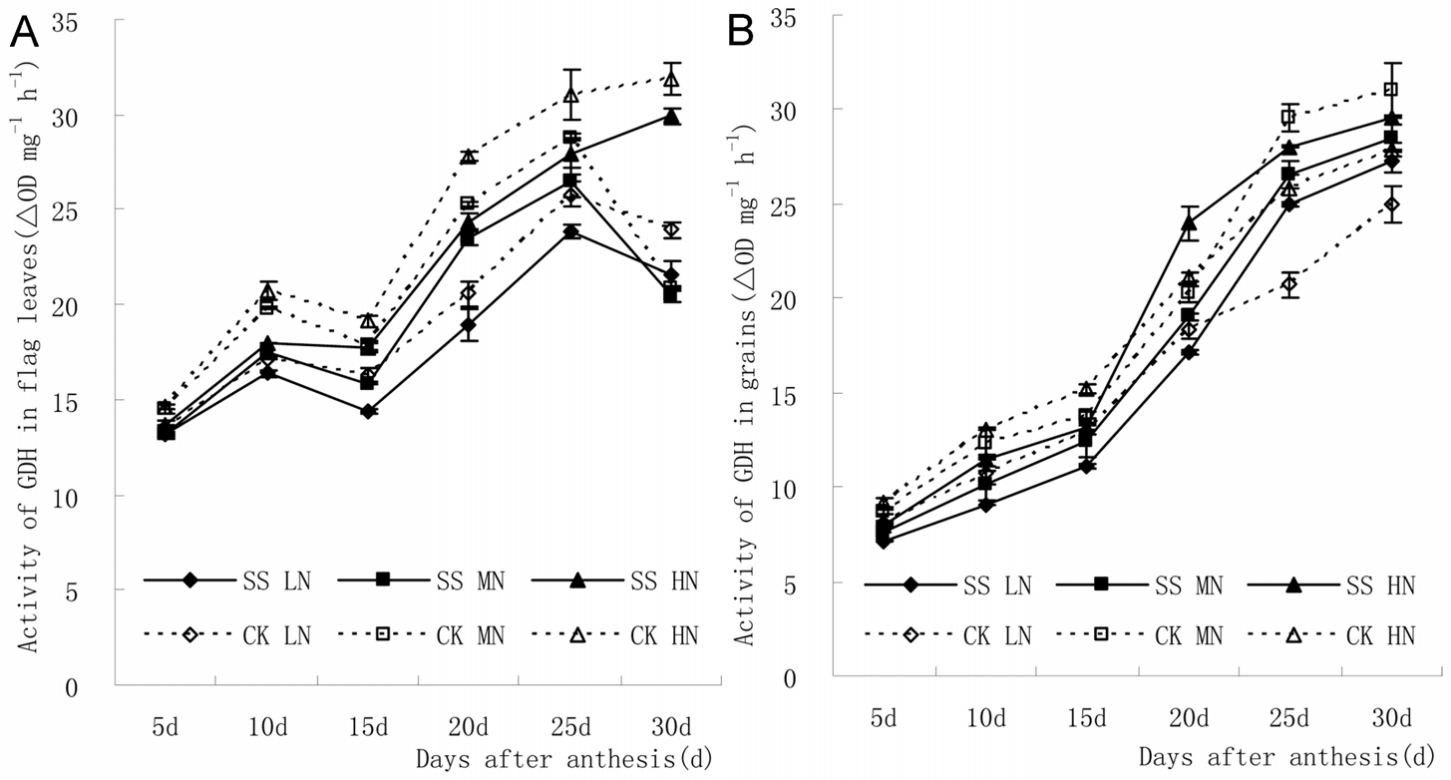
The GDH activity in flag leaves after anthesis increased at first and then decreased, and then increased again (A). In grains, GDH activity kept increasing after anthesis, and had a rapid increment between 15–20 d after anthesis (B).

Fig. 4 displays the change tendency of GS activity in flag leaves and grains after anthesis. Generally, the GDH activity in flag leaves after anthesis increased at first and then decreased, and then increased again. The change of GDH activity influenced by N application was not consistent at 25–30 d after anthesis. In grains, GDH activity kept increasing after anthesis, and had a rapid increment between 15–20 d after anthesis.

Fig. 4A showed subsoiling in fallow period decreased GDH activity in flag leaves after anthesis, and the differences were statistically significant at 5–20 d after anthesis. Besides, the increment of N application increased GDH activity in flag leaves at 10–25 d after anthesis, and the difference between low N application and other treatments was significant. In addition, the regulatory effect of N application on GDH activity in flag leaves was more significant in subsoiling plot than that in no-tillage plot.

Fig. 4B showed subsoiling in fallow period could decrease GS activity in grains at 5–20 d with low N application, 5–30 d with medium N application, and 5–15 d with high N application. The differences of GDH activity between subsoiling plots and no-tillage plots were statistically significant at 5–25 d after anethesis. Increment of N application could increase GDH activity in grains under the condition of subsoiling, and difference between high N application treatment and low N application treatment were significant. In addition, increment of N application could also increase GDH activity in grains at 5–20 d after anthesis in the no-tillage plot, and the difference at 5 d and 15 d after anthesis were significant. In addition, the regulatory effect of N application on GDH activity in grains was more significant in subsoiling plot than that in no-tillage plot at the late milking stage.

### Effects of Subsoiling in Fallow Period on Protein Content and its Component in Grains and its Regulatory Effect by Nitrogen Application

Table 3 showed that subsoiling in fallow period significantly decreased the content of albumin and gliadin in grains. The total protein content was also decreased by subsoiling in fallow period and the difference was statistically significant in the low and medium N application treatments. However, globulin content, Glu/Gli (the ratio beween Glutelin/Gliadin), and protein yield were significantly increased. Glutelin content was also increased by subsoiling in fallow period and the difference in high N application were significant. Table 3 also indicated the increment of N application could significantly increase the content of albumin, glutelin and total protein. It also increased globulin content and protein yield, and the difference between low N application and other treatments were significant under subsoiling condition. Gliadin content in high N application treatment was significantly higher than that in the other treatments, while difference between the other treatments was not significant. Glu/Gli in medium N application was significantly higher than that in the other treatments under the condition of subsoiling (Table 3).

**Table 3:** Effect of subsoiling in fallow period on grain protein and its component contents.

Tillage	applicationamount	Albumin	Globulin	Gliadin	Glutenin	Glu/Gli	Protein	Protein yield
		(%)	(%)	(%)	(%)		(%)	(kg/hm^2^)
SS	LN	2.43c	1.34b	3.39b	3.97c	1.17b	12.30c	506.95b
	MN	2.53b	1.39a	3.33b	4.21b	1.26a	12.97b	578.60a
	HN	2.69a	1.41a	3.75a	4.56a	1.22b	14.19a	597.81a
CK	LN	2.52c	1.22b	3.65b	3.89c	1.07b	13.08c	423.81b
	MN	2.68b	1.27ab	3.68b	4.14b	1.13a	13.51b	498.33a
	HN	2.86a	1.31a	3.97a	4.38a	1.10a	14.43a	506.64a

The difference between data with different letters from a to c are statistically significant (P<0.05).

SS: subsoiling; CK: no tillage; LN: low nitrogen application; MN: medium nitrogen application; HN: high nitrogen application.

### Correlation Analysis between Soil Water Storage and Grain Protein Accumulation in Dryland Wheat

#### Correlation analysis between soil water storage and proline content after anthesis

Table 4 showed soil water storage in the 0–300 cm depth was in negative correlation to proline content in flag leaves and grains. The correlation between soil water storage in the 40–160 cm depth and proline content in grains was significant, while the correlation between water storage in the 0–300 cm depth soil and proline content in flag leaves was not significant. Table 4 also indicated that proline content in grains was significantly correlated with soil water storage in the two layers of 0–100 cm and 100–200 cm, and the latter correlation was more significant. However, the correlation between water stage in 200–300 cm depth soil and proline content was not significant (Table 4).

**Table 4:** Correlation coefficients between soil water storage at different depths and proline content in anthesis.

Soil depth	Flag leaves	Grains
20	−0.4820	−0.5408
40	−0.6424	−0.8550*
60	−0.4059	−0.7612*
80	−0.5719	−0.8299*
100	−0.5087	−0.8225*
0–100	−0.5647	−**0.8224** *
120	−0.5935	−0.8182*
140	−0.6591	−0.8494*
160	−0.4522	−0.7615*
180	−0.4847	−0.7504
200	−0.4081	−0.7268
100–200	−0.5212	−**0.7909** *
220	−0.7360	−0.7367
240	−0.6631	−0.6878
260	−0.6207	−0.5995
280	−0.6702	−0.5518
300	−0.6296	−0.6225
200–300	−0.6660	−**0.6363**

* P<0.05.

#### Correlation analysis between proline content and activity of nitrogen metabolism enzyme after anthesis

Table 5 showed proline content in flag leaves was in significantly positive correlation to GS and GDH activity in flag leaves after anthesis while its correlation to GS and GDH activity in grains was not significant. Proline content in grains was in significantly positive correlation to GS and GDH activity in flag leaves after anthesis while its correlation to GS and GDH activity in grains was not significant. In addition, the correlation between proline content and GDH activity was a bit higher than that between proline content and GS activity (Table 5).

**Table 5:** Correlation coefficients between proline content and activities of the relevant enzymes for protein synthesis.

Proline content	GS activity		GDH activity	
	Flag leaves	Grains	Flag leaves	Grains
Flag leaves	0.7911*	−0.3145	0.8493*	0.5473
Grains	0.9418**	0.1970	0.9845**	0.7130

* P<0.05;

** P<0.01.

#### Correlation analysis between grain protein accumulation and proline content as well as activity of nitrogen metabolism enzyme after anthesis

Table 6 showed contents of albumin, gliadin and total protein in grains were all in positive or significantly positive correlation to proline content in grains and proline content, GS activity and GDH activity in flag leaves. The correlation to proline content in grains was slightly higher than that in flag leaves. The correlation to GDH activity in flag leaves was slightly higher than GS activity. Content of globulin and glutenin together with protein yield were all in positive correlation or significantly positive correlation to GS activity in grains. In addition, content of albumin, glutenin and total protein were in significantly positive correlation to activity of GDH in grains (Table 6).

**Table 6:** Correlation coefficients between content of protein and its components, protein yield and proline content, activity of relevant enzymes for protein synthesis in grains.

protein content and yield	Proline content		GS activity		GDH activity	
	Flag leaves	Grains	Flag leaves	Grains	Flag leaves	Grains
Albumin content	0.8657*	0.9658**	0.9787**	0.1591	0.9918**	0.8239*
Globulin content	−0.4815	−0.0107	0.1366	0.9443**	0.0495	0.2784
Gliadin content	0.9274**	0.9610**	0.8449*	−0.0168	0.9112**	0.6003
Glutenin content	0.2782	0.6925	0.7479	0.7949*	0.7316	0.7945*
Protein content	0.7673*	0.9632**	0.9070**	0.3441	0.9677**	0.7991*
Protein yield	−0.3623	0.0842	0.2466	0.9123**	0.1653	0.4798

* P<0.05;

** P<0.01.

## Discussion

In recent years, dryland cultivators have conducted many researches to study the water storage and retention of dryland wheat, and great progress had been achieved [5]–[8]. Many results [21]–[29] showed no-tillage or little tillage had a clear effect on soil water retention. Zhao et al. (2007) stated continuous tillage would form water exclusion in the bottom of harrow which was unfavorable to water storage of soil and the ability of water retention was also limited [9]. However, Zhu and Jia (1997) stated that hydraulic conductivity of soil was improved under protected cultivation, thus water infiltration would increase and massive water loss during plowing would be decreased which result in improving efficiency and availability of water [21]. Besides, Xu et al. (2000) studied effect of different tillage method (conventional tillage, no-tillage, and subsoil tillage) on physical properties and hydraulics of soil. And their results showed subsoiling significantly decreased volume weight of soil and increased porosity of soil and improved the conductivity of soil water. On the other hand, Wang et al. (2006) stated that subsoiling could break the limitation of root system, improve the depth of root system distribution and the efficiency of water use [24].

In agreement with previous studies, our results also showed that subsoiling (depth 30–40 cm) significantly increase water storage and retention (Fig. 1). Subsoiling can break the plow pan of the farmlands in dry-farming areas which may accelerate the infiltration of rainwater. Additionally, it is easy for plant roots to grow into the deep layer in the loose soil created by subsoiling and the developed root system probably help to hold the rainwater and increase water storage. The different results obtained by researchers might attribute to the varied soil and climate conditions in different area, that is, soil porosity, amount of precipitation, wind-force and temperature, etc. The experimental site which has a relative higher precipitation and lower evaporation rate may result in high water storage and retention after subsoiling.

The present experiment was conducted on a field which was no-tillage in fallow period for many years and performed rotary tillage before sowing. Subsoiling in fallow period broke the bottom of plough layer formed many years which was crucial to water storage and retention, and could increase water storage of 0–300 depth soil at each stage. Especially, the increment in the 60–160 cm depth amounted to more than 10% of the total water storage. It also increased water storage in the 0–200 cm depth soil with low and medium N application until anethesis. However, water storage in the 0–200 cm depth with high N application decreased which was possibly caused by great water consumption due to high biomass of plants. In addition, whether the dryland wheat was subsoiled in fallow period affected the regulatory effect of N application. Under the condition of subsoiling, N application obviously regulated the soil layer with the depth of 40 cm, 60 cm and 120–200 cm. Water storage of soil in the 200–300 cm depth was the least when N application was 150 kg·hm^−2^. Accordingly, subsoiling in fallow period together with N application at 150 kg·hm^−2^ were beneficial to root downward growth and absorption of water in deep soil.

As subsoiling breaking the plough layer and loosing the soil, fertilizer could also easily reach to the deep soil and used by the plant root in the same layer. And this may heighten the utilization of N to synthesize protein/amino acids and lead to a better wheat productivity. On the other hand, the developed root system would accelerate the absorption of water and nutrition which result in the flourish of wheat leaves. Thus, the photosynthesis is enhanced and more biomass would be accumulated together with a high grain yield.

Osmoregulation is an important mechanism of plant adapting to drought [30], which contributes to continual water adsorption of water from environment with declining water potential and prevents dehydration. Proline plays an important role in osmoregulation [31]. Proline content in the plant is normally low, but it will increase to tenfold under the condition of drought which accounting for more than 30% of total free amino acids [32]. In higher plant, there are two ways to synthesize proline, and both of them are from transformation of glutamic acid which is closely relevant with nitrogen metabolism. Assimilation of ammonia is mainly realized by the circulation of GS/GOGAT and an alternate way is the reaction catalyzed by NADH-GDH which is dependent on reductive coenzyme I. This is a compensation to the circulation of GS/GOGAT under certain physiological conditions [33]. Research on tobacco showed that GS played a main role in synthesis of proline under drought while GDH played an important role in providing glutamic acid for synthesis of proline under salty [34]. Study on wheat showed that GS and GDH had different contributions to proline accumulation in wheat seedlings under salty condition. Under low concentration of salt stress, GS played a major role in synthesis of glutamic acid and GDH played a major role in providing precursor for synthesis of proline under high salt stress [35]. The roles of GS in drought stress have been studied in tobacco and rape but the study on the role of GDH in synthesizing proline under drought is lacking. Our study showed that drought in anthesis increased proline content in grains of dryland wheat. GS and GDH in flag leaves both played important roles in synthesis of grain proline and GDH was much more important. In addition, contents of albumin, gliadin and total protein were considerably relative to proline content in grains and GDH activity in flag leaves. Therefore, subsoiling in fallow period decreased contents of albumin, gliadin and protein in grains.

## Conclusion

Our study showed subsoiling could increase glublin content, Glu/Gli, protein yield, glutelin content. Grain protein is an important index to evaluate the quality of wheat. Although subsoiling decreased total protein content, the ratio between glutelin and gliadin was increased. Hence, the quality was improved. And the maximum value was obtained under N application 150 kg·hm^−2^. In summary, subsoiling in fallow period was proved as an effective technology of water storage and retention. In addition, it was more favorable to improve the quality of dryland wheat grain accompanied with an N application of 150 kg·hm^−2^.
